# Application of the RBBP9 Serine Hydrolase Inhibitor, ML114, Decouples Human Pluripotent Stem Cell Proliferation and Differentiation

**DOI:** 10.3390/ijms21238983

**Published:** 2020-11-26

**Authors:** Seakcheng Lim, Rachel A. Shparberg, Jens R. Coorssen, Michael D. O’Connor

**Affiliations:** 1 School of Medicine, Western Sydney University, Campbelltown NSW 2560, Australia; s.lim@centenary.org.au (S.L.); R.Shparberg@westernsydney.edu.au (R.A.S.); 2 Departments of Health Sciences and Biological Sciences, Faculties of Applied Health Sciences and Mathematics & Science, Brock University, St. Catharines, ON L2S 3A1, Canada; jcoorssen@brocku.ca; 3 Molecular Medicine Research Group, Western Sydney University, Campbelltown NSW 2560, Australia

**Keywords:** human pluripotent stem cells, ML114, NFYA, pluripotency, proliferation, RBBP9, bioinformatics

## Abstract

Retinoblastoma binding protein 9 (RBBP9) is required for maintaining the expression of both pluripotency and cell cycle genes in human pluripotent stem cells (hPSCs). An siRNA-based study from our group showed it does so by influencing cell cycle progression through the RB/E2F pathway. In non-pluripotent cells, RBBP9 is also known to have serine hydrolase (SH) activity, acting on currently undefined target proteins. The role of RBBP9 SH activity in hPSCs, and during normal development, is currently unknown. To begin assessing whether RBBP9 SH activity might contribute to hPSC maintenance, hPSCs were treated with ML114—a selective chemical inhibitor of RBBP9 SH activity. Stem cells treated with ML114 showed significantly reduced population growth rate, colony size and progression through the cell cycle, with no observable change in cell morphology or decrease in pluripotency antigen expression—suggesting no initiation of hPSC differentiation. Consistent with this, hPSCs treated with ML114 retained the capacity for tri-lineage differentiation, as seen through teratoma formation. Subsequent microarray and Western blot analyses of ML114-treated hPSCs suggest the nuclear transcription factor Y subunit A (NFYA) may be a candidate effector of RBBP9 SH activity in hPSCs. These data support a role for RBBP9 in regulating hPSC proliferation independent of differentiation, whereby inhibition of RBBP9 SH activity de-couples decreased hPSC proliferation from initiation of differentiation.

## 1. Introduction

The retinoblastoma (RB) binding protein 9 (RBBP9—previously termed Bog [1,2] and RBBP10 [3]) is expressed in human pluripotent stem cells (hPSCs) and in a range of human cancer cells [1,4,5,6]. However, little is known of the roles RBBP9 plays either in normal development or cancer progression. A small number of studies have suggested RBBP9 has two different activities: (i) the ability to bind RB protein and regulate the activity of the RB/E2F cell cycle pathway [1], and (ii) the ability to act as a serine hydrolase (SH) on as yet undefined target proteins [4]. In terms of RB-binding, RBBP9 has been shown to displace E2F1 from RB/E2F1 complexes, allowing expression of cell cycle related genes [1,4]. In contrast, the SH activity of RBBP9 is more poorly understood. Comparison of protein sequences identified a conserved serine residue hypothesized to be the putative nucleophilic serine (Ser75) within a GXSXG motif common to other SHs [4,7,8,9]. This finding is supported by X-ray crystallography that suggests RBBP9 is a member of the ‘domain of unknown function’ superfamily (DUF1234) of serine proteases [7]. Overexpression of the catalytically inactive mutant RBBP9-S75A in human carcinoma cells demonstrated that loss of RBBP9 SH activity resulted in decreased proliferation [4]. While a number of these studies made use of commercially available SH probes, the lack of specificity of these probes makes them poorly suited to assessing RBBP9 SH activity in a cellular context where other SH enzymes are expressed (such as hPSCs).

High throughput screening for inhibitors of enzymes with poorly characterized biochemical activity identified a potent and specific chemical inhibitor of RBBP9 SH activity—ML114 [10]. The study showed that the cell-free IC50 for ML114 on recombinant RBBP9 is 0.63 μM. In comparison, the IC50 for ML114 on 30 other SH was found to be >100 μM. At 20 to 100 μM, ML114 blocked the SH activity of recombinant RBBP9 in human embryonic kidney 293T (HEK) cells, as well as RBBP9 doped into the membrane protein fraction of mouse brain [11]. Notably, the cellular toxicity associated with ML114 is low, with its CC50 being >100 μM in HEK cells [10].

In a study aimed at better understanding the molecular circuitry of pluripotency, RBBP9 was identified as a novel regulator of human pluripotent cells [12]. That study showed loss of RBBP9 mRNA and protein expression—induced by RBBP9-specific small interfering RNA (siRNA)—disrupted key pluripotency-related properties including: (1) reducing the number of pluripotent cells, as detected by the colony forming cell (CFC) assay; (2) decreasing FOXD3 expression; (3) decreasing cell cycle gene expression; and (4) increasing expression of neural differentiation genes. These data suggest RBBP9 plays a role in maintaining molecular networks required for pluripotency and inhibition of differentiation. However, the siRNA approach used in that study did not determine the relative contribution of RB-binding and SH activities to hPSC maintenance. More detailed analysis of the role of RBBP9 in hPSC maintenance and early development could provide useful information on how pluripotent cells are generated or maintained, and on mechanisms of both normal and cancer development.

As a first step towards understanding the relative importance of RBBP9 SH activity in hPSC maintenance, the present study assessed the effects of treating hPSCs with the RBBP9 SH inhibitor ML114. Interestingly, ML114 treatment partially phenocopied the published effects of siRNA-mediated loss of RBBP9 protein, though with a key difference: ML114-treated hPSCs showed a reduced population growth rate and altered proliferation dynamics, but the treated hPSCs retained pluripotency marker expression and teratoma-forming ability. These data suggest that ML114 treatment decouples hPSC differentiation from inhibition of hPSC proliferation—an unusual, though not unprecedented, phenomenon. Moreover, these data suggest it is the RB-binding activity of RBBP9—not the SH activity—that impacts on hPSC differentiation. Further investigation and clarification of RBBP9 and its activities will enable a greater understanding of its role in the maintenance of hPSCs, in development, and in the progression of cancer.

## 2. Results

### 2.1. ML114 Reduces hPSC Population Growth Rate without Inducing Differentiation

**Figure 1:**
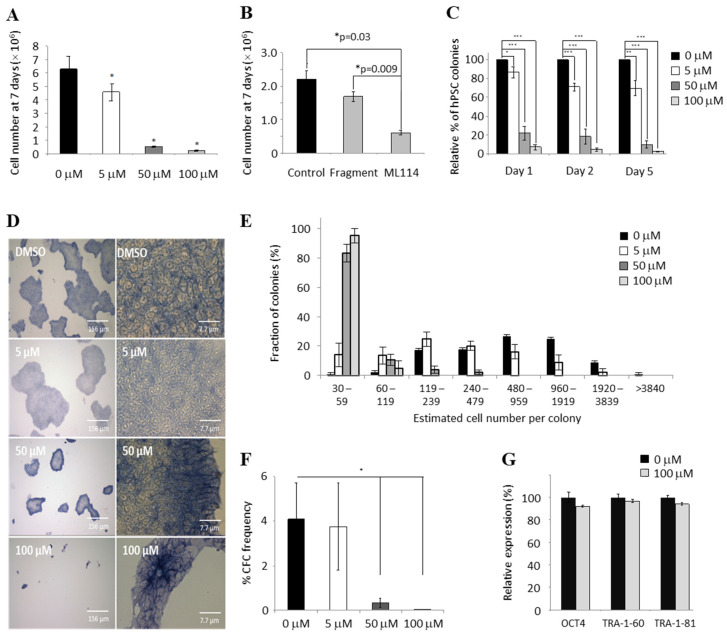
ML114 reduces hPSC growth rate without reducing pluripotency markers. (**A**) Dose response data demonstrating higher concentrations (50–100 µM) of ML114 reduced hPSC yield after 7 days of treatment (*n* = 3; * *p* < 0.05). (**B**) The hPSC population size was significantly reduced after 7 days of 100 μM ML114 treatment, whereas no significant change in the hPSC population was caused by treatment with the soluble ML114 fragment at 100 μM (Fragment; *n* = 3; *p* > 0.05). (**C**) ML114 treatment decreased the number of CFCs that attached during the first 24 h (i.e., Day 1 data; *n* = 5; * *p* ≤ 0.05, ** *p* ≤ 0.001, *** *p* ≤ 0.0001). However, the CFCs that did attach remained attached for the duration of the assay—as shown by the similar frequency of colonies on Day 2 and Day 5 compared to Day 1. (**D**) Smaller colonies were seen with increasing concentrations of ML114. However, no change in cell morphology was caused by the ML114 treatment, nor was there any reduction in the level of alkaline phosphatase staining (*n* = 5). (**E**,**F**) Increasing concentrations of ML114 reduced the size of the colonies detected on Day 7 of the CFC assay, ((**E**); *n* = 5; * *p* ≤ 0.05). Additionally, increasing concentrations of ML114 reduced the number of CFCs (**F**). (**G**) Flow cytometry revealed control- and ML114-treated hPSCs express similarly high levels of pluripotency associated antigens OCT4, TRA-1-60, and TRA-1-81 (*p* > 0.05 and *n* = 3 for each antigen and treatment).

To investigate whether endogenous, physiologically-relevant levels of RBBP9 SH activity might be involved in maintenance of the pluripotent state, hPSCs were initially cultured with the small molecule ML114. This molecule was chosen as it selectively inhibits RBBP9 SH activity with high specificity, but without inhibiting thirty other SHs—for example, when used at 200 μM in mouse brain membrane proteome extracts [11]. An initial dose-response experiment was therefore performed here to assess the effects of ML114 in hPSCs (Figure 1A). Vehicle-control (0.25% DMSO) was compared with increasing concentrations of ML114 up to 100 μM—the highest reported concentration enabling inhibition with minimal cell death due to toxicity [11]. All concentrations of ML114 tested significantly reduced the hPSC population growth rate after 7 days of culture—as shown by reduced cell numbers compared to the control treatment (Figure 1A). The greatest effect was seen with 100 μM ML114. To test whether this effect was due to the release/presence of the ML114 cleavage product (termed “ML114 fragment”), hPSCs were cultured with only the cleavage product. Compared to the control treatment, no significant change in hPSC numbers was seen when the ML114 fragment was included in the cultures up to 100 μM (Figure 1B; *p* > 0.05). These data show that treatment with ML114—but not its RBBP3 SH cleavage product—decreases the hPSC population growth rate, suggesting inhibition of RBBP9 SH activity might be responsible for these effects.

### 2.2. ML114 Reduces Pluripotent CFC Number and Colony Size without Inducing Differentiation

As inhibition of hPSC proliferation has been correlated with hPSC differentiation [12,13,14], various pluripotency assays were performed to assess whether ML114 treatment induces differentiation in addition to its effect on the hPSC population growth rate. Application of the alkaline phosphatase-based CFC assay—a sensitive indicator of hPSC numbers [15]—showed a decrease in the colony number within the initial 24 h, but no further decrease was observed over subsequent days (Figure 1C). In addition, ML114 treatment did not significantly alter hPSC morphology or decrease expression of the pluripotency marker alkaline phosphatase—indicating no initiation of differentiation (Figure 1D). These data suggested ML114 might impair hPSC attachment and/or cell survival within the initial cell-seeding phase of the assay. However, assessment of dead cell numbers between one and five days after treatment showed no significant difference between the treatments at any of the timepoints (Supplementary Figure S1A).

To assess whether ML114 reduced the growth rate of the colonies derived from the CFCs, the number of cells per colony was determined for each treatment. The CFCs exposed to higher concentrations of ML114 had fewer larger colonies (Figure 1E), and fewer total numbers of pluripotent CFCs (Figure 1F). Control- and ML114-treated hPSCs were also assessed via flow cytometry, to look for changes in expression of the pluripotency antigens OCT4, TRA-1-60, and TRA-1-81. This analysis revealed equally high levels of all three pluripotency-associated antigens in both control- and ML114-treated hPSCs—even after 7 days of treatment with 100 μM of ML114 (Figure 1G; Supplementary Figure S1B). Collectively, these flow cytometry and CFC assay data show that ML114 treatment decreases both the frequency and proliferative capacity of pluripotent colony-forming cells, without reducing the expression of a range of intracellular or extracellular antigens associated with pluripotency.

### 2.3. ML114 Slows Progression from G_0_/G_1_ into S-Phase without Differentiation or Karyotype Changes

**Figure 2:**
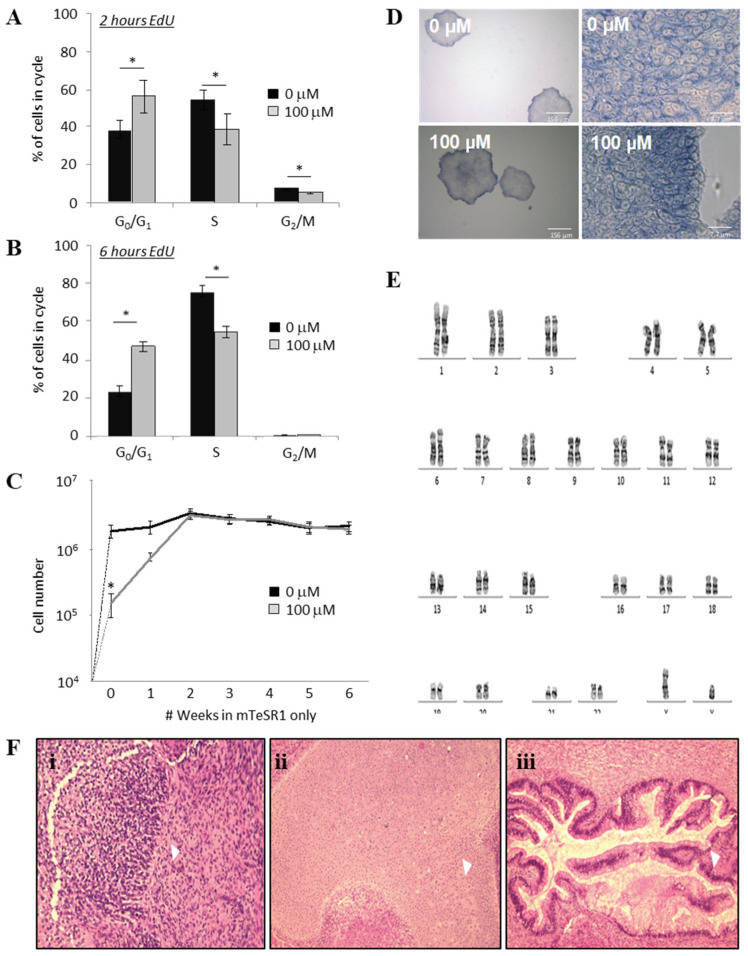
ML114-treated hPSCs proliferate more slowly, retaining pluripotency without differentiating. (**A**,**B**) Flow cytometry assessment of control- and ML114-treated hPSCs exposed to EdU for (**A**) 2 h and (**B**) 6 h. The ML114-treated hPSCs have significantly fewer EdU^+^/S-phase cells at both time-points: by 2 h ~55% of control-treated cells were EdU^+^, whereas the ML114-treated cells required 6 h to reach this same number of Edu^+^ cells (*n* = 4 per treatment; * *p* < 0.05). (**C**) hPSC numbers in control- and 100 μM ML114-treated cultures assessed following removal of treatment (at Week 0) and weekly reseeding in mTeSR1 (*n* = 4; * *p* < 0.05). (**D**) CFC assay data from hPSCs previously treated with ML114. Cells in the resulting colonies display identical cell morphology, colony size and alkaline phosphatase staining compared to the CFC assay data from control-treated hPSCs. (**E**) Karyotype data show hPSCs exposed to 100 μM ML114, followed by a further 11 weeks of culture in standard maintenance media only, do not possess abnormal chromosomes at a resolution of 400 bands per haploid set. (**F**) Haematoxylin and eosin-stained sections from teratomas derived from hPSCs treated for 7 days with 100 mM ML114 then cultured in standard maintenance conditions for 11 weeks. All 3 germ layers are present in the teratomas including: ectoderm (e.g., neuroepithelium: i, arrowhead), mesoderm (e.g., stroma, cartilage, and smooth muscle: ii, arrowhead) and endoderm (e.g., glandular epithelium: iii, arrowhead).

To test whether the reduced hPSC population growth rate, reduced CFC colony numbers and reduced colony size caused by ML114 were due to cell cycle changes, both control-and ML114-treated cells were assessed using the EdU cell proliferation assay. Cells were assessed both 2 and 6 h after the ML114 treatment. The control-treated cells had a high proliferation rate consistent with that reported for human pluripotent cells [13]. However, both the 2-h (Figure 2A) and 6-h (Figure 2B) EdU data showed ML114-treated hPSCs progressed through the cell cycle significantly more slowly. After 2 h of EdU exposure approximately 55% of control hPSCs had entered the S-phase (Figure 2A). In contrast, it took 6 h for the same number of ML114-treated hPSCs to enter the S-phase (Figure 2B). These data indicate ML114 causes hPSCs to progress through the cell cycle approximately three times slower than control-treated hPSCs. Based on this difference in proliferation, together with the input seeding density and the attachment rate in the presence of ML114 (Figure 1C), the control treatment was predicted to produce approximately 7.5 million cells and the ML114 treatment was predicted to produce approximately 150,000 cells—consistent with the data obtained in Figure 1A).

### 2.4. The Effects of ML114 on hPSCs are Reversible

To test whether the effects of ML114 were reversible, hPSCs were cultured with control- or ML114-treatment for 7 days before being re-seeded into optimal hPSC maintenance conditions and cultured without any treatment for another 5 weeks (Figure 2C; weeks 1–6). As described above, ML114 treatment resulted in a dramatic reduction in hPSC numbers by the end of the 7-day treatment period (Figure 2C). However, after removal of ML114, the growth rate of the ML114-treated hPSCs increased to become indistinguishable from that of the control cultures within approximately 2 weeks (Figure 2C). Additionally, CFC assays performed at the end of the 6-week period, on the cultures derived from the control- and ML114-treated cells, were indistinguishable (Figure 2D).

### 2.5. ML114 Does Not Induce Genomic Instability or Remove Differentiation Capacity

To assess whether the reduced growth rate and reduced cell cycling of the ML114-treated hPSCs might be associated with karyotypic abnormalities, the ML114-treated cells were analyzed via G-banding. Assessment of 15 metaphases (at a resolution of 400 bands per haploid set) from ML114-treated cultures showed no chromosomal abnormalities (Figure 2E).

To determine whether the ML114-treated hPSCs retained multi-lineage differentiation capacity, they were assessed using the teratoma assay. Cells were treated with 100 μM ML114 for 1 week. The treated cells were then expanded in standard pluripotency-maintenance conditions to assess whether any effects of ML114 treatment required more time to appear. As no changes were noted the teratoma assay was performed. The progeny of the ML114-treated cells were shown to retain the ability to form teratomas containing cell types representative of endoderm, mesoderm, and ectoderm (Figure 2F).

### 2.6. hPSC-Expressed Genes Affected by ML114 Treatment Are Involved in Protein Modification Processes

To begin identifying the molecular consequences of ML114-treatment, Affymetrix gene expression analysis was performed in triplicate on control- and 100 μM ML114-treated hPSCs. This analysis detected less than 1.5-fold upregulation of 3107 transcripts (2208 protein-coding genes) in the ML114-treated samples (*p* < 0.002, false-discovery rate of 0.021; Supplementary Table S1). This included the pluripotency regulator, NANOG, and cell cycle genes *CCNB2*, *CDC25C* and *CDK5*. Interestingly, no genes were significantly decreased in expression as a result of ML114 treatment. This suggests that the decrease in expression of *FOXD3*—whose promoter contains binding sites for E2F1 to E2F5—caused by RBBP9 siRNA, is due to loss of RBBP9’s RB-binding activity causing release of RB and subsequent inactivation of E2F transcription factors. Similarly, this Affymetrix data suggests that the increase in neurogenesis genes that occurred with RBBP9 siRNA treatment was due to loss of RB-binding activity, rather than loss of SH activity.

Gene ontology analysis of the 2208 ML114-up-regulated genes was performed to examine what biological processes these genes might perform. This analysis showed only one GO term was significantly enriched, “protein modification processes” (Supplementary Table S2). Included in this category were genes involved in cell cycle regulation, proteolysis and apoptosis (Supplementary Tables S3–S5). A number of these genes increased by ML114 treatment are known targets of transcription factors involved in regulation of proliferation and PSC maintenance. For example, CDC25C and CCNB2 are targets of NFYA [16,17]. No significant enrichment was found for GO terms related to differentiation.

### 2.7. NFYA Is a Predicted Target of RBBP9 SH Activity

To identify candidate transcription factors responsible for the gene expression changes caused by ML114, promoter analyses were performed on the differentially-expressed genes to look for common DNA-binding motifs. Two separate promoter analyses were performed on the ML114-regulated genes: (i) the proximal analysis interrogated a region ±400 bp on either side of the transcription start sites; and (ii) the distal analysis interrogated a region 10 kb upstream from the transcription start sites. Using this approach, the transcription factors DEAF1 and NFYA were highly ranked as regulators of the ML114-affected genes in both the proximal and distal analyses (Table 1 and Table 2). These two transcription factors were implicated in proliferation, PSC maintenance, and early embryo development [16,18,19,20].

Comparison of the 2208 ML114-regulated genes with published gene expression data from RBBP9 siRNA-treated hPSCs [12] identified 152 genes up-regulated by both RBBP9 siRNA and ML114. Promoter analyses of these 152 genes were also performed (Table 3 and Table 4). Transcription factors identified in this way included known regulators of pluripotent cells. For example, SP1, previously implicated in tumorigenesis and cell cycle regulation [21,22,23], and as a regulator of NANOG expression in murine embryonic carcinoma cells [24]. Notably, DEAF1 (proximal analyses) and NFYA (CBF_01; distal analysis) were again predicted as regulators of these 152 RBBP9-regulated genes.

### 2.8. Western Blot Analysis of ML114-Treated hPSCs Supports NFYA as an Effector of RBBP9 SH Activity

To test whether DEAF1 or NFYA levels in hPSCs might be affected by ML114 treatment, both gene and protein expression analyses were performed. Analysis of the Affymetrix gene expression data showed no increase in *DEAF1* mRNA levels between DMSO-, ML114 fragment- and ML114-treated hPSCs (Supplementary Figure S2A). Similar results were obtained for DEAF1 protein via Western blotting (Supplementary Figure S2B).

**Figure 3:**
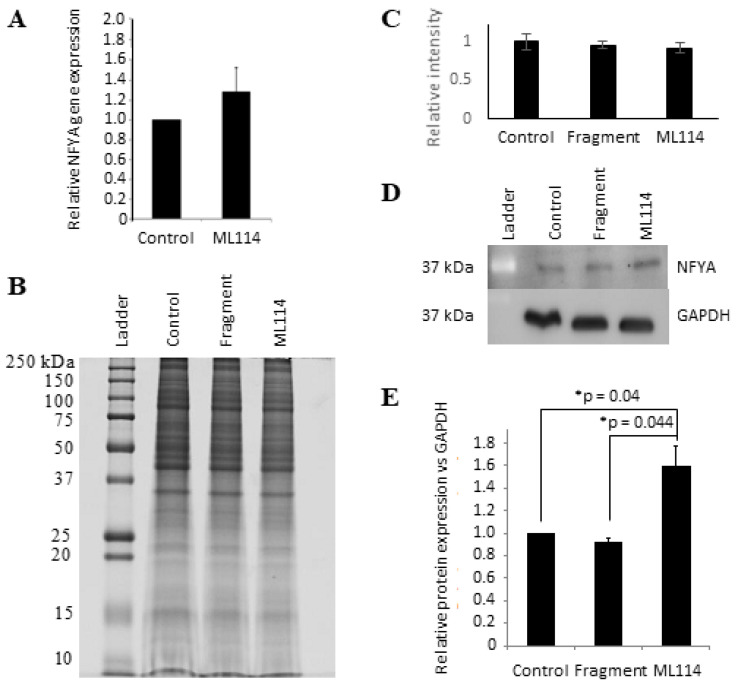
NFYA protein expression is significantly increased in ML114-treated hPSCs. (**A**) Real-time PCR data showing no significant increase in *NFYA* transcript expression caused by 100 μM ML114 (*p* = 0.39). (**B**,**C**) Image of a Coomassie blue-stained SDS-PAGE gel (**B**) and quantification of whole-lane protein staining (**C**) show similar gross-protein expression patterns in hPSCs treated with: Control (DMSO-only); 100 μM ML114 fragment (“Fragment”); and 100 μM ML114 (“ML114”). (**D**,**E**) Western blot (**D**) and associated densitometry quantification (**E**) showing a significant increase in NFYA protein levels in ML114-treated hPSCs compared to the control treatments (*n* = 3 for all treatments).

For *NFYA*, the Affymetrix data showed no significant difference in transcript levels between DMSO- and ML114-treated hPSCs, and this was confirmed using real-time PCR (Figure 3A). In contrast, Western blot showed a statistically-significant increase of ~50% in NFYA protein levels in the hPSCs treated with 100 μM ML114 (Figure 3B–E)—suggesting NFYA might be either a direct or indirect target of RBBP9 SH activity.

## 3. Discussion

The protein RBBP9 has two proposed mechanisms of action; i) RB-binding activity, and ii) SH activity [1,4,5]. Both these activities have been shown to regulate cell cycle progression. The RB-binding activity is thought to regulate the cell cycle by binding RB protein, thereby releasing E2F transcription factors required for cell cycle progression [1]. Similarly, loss of RBBP9 SH activity has been shown to decrease proliferation in human pancreatic carcinoma cells [4]. Recently, RBBP9 was identified as a candidate regulator of hPSC maintenance: siRNA-mediated loss of RBBP9 protein decreased hPSC growth rate, down-regulated expression of pluripotency-associated markers, decreased expression of some cell cycle genes, and increased expression of genes associated with neural differentiation [12]. However, that study did not investigate the relative contributions of RB-binding and SH activities to the regulation of pluripotency in hPSCs.

For the present study, we used a specific chemical inhibitor of RBBP9 SH activity—ML114 [10]—to begin investigating the role RBBP9 SH activity might playing in hPSCs. Upon cleavage by RBBP9, a fragment of ML114 covalently binds to the catalytic Ser75 of RBBP9, thereby catalytically-inactivating RBBP9 SH activity. At the same time, a small fragment of the ML114 is released [10]. ML114 inhibits RBBP9 SH between 20 and 100 μM—concentrations at which ML114 displays low cytotoxicity and at which ML114 does not affect the activity of thirty other serine proteases [11]. Here, use of 100 μM ML114 did not affect phenotypic or functional markers of pluripotency, but ML114 did decrease key properties associated with hPSC proliferation—i.e., population growth rate, CFC frequency, colony size, the proportion of hPSCs in S-phase, and the proportion of cells in G_2_/M. In contrast, treatment with the 100 μM ML114 cleavage fragment did not affect any of these hPSC properties.

### 3.1. Inhibition of RBBP9 SH Activity Decouples Decreased hPSC Proliferation from Differentiation

Numerous studies have shown PSC differentiation is coupled with decreased proliferation [13,14,25], with the G_1_ checkpoint found to act as a control point for initiation of differentiation and lineage commitment [13,14,25,26,27]. Rapid transition from G_1_ to S-phase has been used as an indicator of pluripotency, where cultures undergoing differentiation accumulate cells in G_1_ and have fewer cells in S-phase. In addition, accumulation of hypo-phosphorylated (i.e., active/E2F-sequestering) RB protein in G_1_ in human embryonic stem cells coincided with loss of pluripotency markers such as OCT4, and the onset of differentiation [26]. Consistent with this, literature reports show hPSC differentiation agents rapidly affect key properties of pluripotent cells [28,29,30]. For example, 5-days of retinoic acid treatment induces: large changes in hPSC morphology; a significant reduction in CFC frequency (~80 fold); and a significant (~5-fold) decrease in the expression of pluripotency markers such as OCT4 [29].

Here, ML114 treatment showed (i) RBBP9 SH activity is required to promote hPSC transition from G_0_/G_1_ to S-phase; and (ii) that inhibiting RBBP9 SH activity has no noticeable effect on hPSC differentiation, at least within the 7-day timeframe studied. Continuous use of ML114 for 7 days did not affect hPSC morphology, alkaline phosphatase activity or expression of classical pluripotency antigens. Interestingly, the effects of ML114 on hPSC proliferation were temporary, and were reversed after withdrawal of ML114—consistent with newly transcribed/translated RBBP9 replenishing the RBBP9 covalently inactivated by the ML114 treatment. Once the ML114 treatment was removed, the growth rate of the treated hPSCs returned to that of a normal population, and the cells could form teratomas containing representatives of endoderm, mesoderm and ectoderm.

These combined findings are consistent with a small number of recent reports that show inhibition of hPSC proliferation does not necessarily induce differentiation. For example, knockdown of E2F2 in human embryonic stem cells reduced proliferation and CFC numbers [31]. The E2F2 knockdown cells accumulated in G_1_ yet maintained expression of pluripotency markers and the ability to differentiate via embryoid bodies. Similarly, depletion of PRMT5 in hPSCs increased the number of cells in G_0_/G_1_ and also reduced the number of cells in G_2_/M—but did not affect expression of pluripotency markers and did not remove the capacity for multi-lineage differentiation via embryoid bodies [32]. The effectors involved in this de-coupling of hPSC differentiation and proliferation are poorly understood, indicating further investigation of the molecular circuits regulated by RBBP9 SH activity may provide valuable information on how proliferation, self-renewal divisions and differentiation are regulated in PSCs.

### 3.2. DEAF1 and NFYA: Pluripotency Regulators as Candidate Effectors of RBBP9 SH Activity

The gene expression profiling of ML114-treated hPSCs suggested NFYA and DEAF1 may regulate the transcription of genes whose expression is increased by both ML114 and/or RBBP9 siRNA. No changes were noted for either of these transcripts in the ML114-treated hPSCs, suggesting RBBP9 does not regulate transcription of these genes. Western blotting analyses showed no change in the DEAF1 protein expression resulting from ML114 treatment. This suggests that if DEAF1 is regulated by RBBP9 SH activity, then it is likely to be an indirect regulation. For example, through RBBP9 SH-mediated proteolysis of kinases, phosphatases or other proteins that post-translationally regulate DEAF1. In contrast, Western blotting showed a significant increase of ~50% in NFYA protein levels with ML114 treatment —suggesting NFYA might be a direct or indirect target of RBBP9 SH activity. In support of this, mass spectrometry analyses of HIV-1 cells by Impens et al. showed NFYA as a putative target of serine hydrolase activity [33].

Both NFYA and DEAF1 are reported to have roles in embryonic development and also self-renewal in various PSCs including hPSCs [18,19,20,34,35,36,37,38,39]. NFYA can act as both a transcriptional activator and repressor, and NFY binding motifs are located in promoters of known hPSC regulatory genes such as OCT4, NANOG and SOX2 [18,40,41,42,43]. NANOG has been shown to interact with NFYA in embryonic stem cells [19,35,36,38] and loss of NFYA has been shown to reduce NANOG expression [19,35,38,44]. Consistent with this, the Affymetrix analyses of ML114-treated hPSCs presented here revealed a small but significant increase in *NANOG* transcripts, and *NANOG* transcripts were also upregulated by RBBP9 siRNA treatment—suggesting loss of RBBP9 SH activity could be the cause of increased *NANOG* mRNA expression in both of these cases (thus warranting future investigations). The expression of other genes known to be regulated by NFYA was also upregulated as a result of ML114 treatment, including *CCNB2*, *CDC25C* and *CDK5* [16,45,46]. NFYA target genes may therefore play a role in the reduced hPSC proliferation and population growth rates that resulted from ML14 treatment. Further investigation of RBBP9, DEAF1, NFYA and their effectors in hPSCs could provide useful insights into the molecular circuitry of pluripotency—with relevance to stem cell maintenance and reprogramming strategies. Given the relatively small fold changes seen here with the differentially-expressed genes—and the fact that transcript levels often do not predict the expression level of the related transcribed protein, let alone active proteoforms—further investigations will benefit from detailed proteomic approaches that quantitatively examine proteoform levels and/or activities.

### 3.3. Summary

Overall, the data presented here show ML114 treatment decreases hPSC proliferation. These findings are consistent with published work that shows inactivation of RBBP9 SH activity via mutation of the active site serine also decreases proliferation. Thus, these data suggest RBBP9 SH activity may help promote hPSC proliferation, while the RB-binding activity impacts on hPSC differentiation. As modulating proliferation is an important step in generating induced pluripotent stem cells [47], modulating RBBP9 SH activity during reprogramming strategies might improve the efficiency of induced pluripotent stem cell production. This is further supported by the finding that the decrease in proliferation caused by ML114 was not associated with induction of hPSC differentiation—thereby providing additional support for the ability to decouple cell cycle inhibition in hPSCs from initiation of differentiation. This phenomenon might also enable the use of ML114 to synchronize stem cell cycling prior to initiation of differentiation, either to investigate how cell cycle regulators and lineage-specific transcription factors are coordinated in hPSCs, or to aid production of desired, differentiated cell types. Further investigation of RBBP9 SH activity in hPSCs might also provide a useful framework for understanding a wide variety of human cancers in which RBBP9 is expressed—including pancreatic, ovary, colon, lung, and breast cancer [4]. For example, increased knowledge of the cell cycle, self-renewal and differentiation networks regulated by RBBP9 SH activity could provide useful insights into the role of RBBP9 in cancer cells, including slowly proliferating cancer cells resistant to current immunotherapies [48].

## 4. Materials and Methods

### 4.1. Cell Culture

CA1 human embryonic stem cells used here were obtained from Professor Andras Nagy from The University of Toronto, Canada. These cells were chosen as extensive characterization by the International Stem Cell Initiative—established to define baseline parameters for human embryonic stem cells—which demonstrated CA1 cells behave the same as other human embryonic stem cells [49]. Use of hPSCs complied with national guidelines with oversight by the Western Sydney University Human Research Committee and the Biosafety and Radiation Committee. CA1 cells were seeded as aggregates (~150,000 cells) or single cells (up to 10,000 cells) using the defined, feeder cell-free medium mTeSR1™ [50] and the extracellular matrix product Matrigel. For treatment with ML114, determination of IC50 and IC90 values using hPSCs was not possible due to the absence of any phenotypic or functional read-outs for RBBP9 serine hydrolase activity in the hPSCs. Therefore, the effect of different doses of ML114 was initially tested on undifferentiated stem cells. The hPSCs were treated either with 0.25% DMSO (Sigma Aldrich, Castle Hill, Australia) as a vehicle-only control, the ML114 soluble fragment (Key Organics) in 0.25% DMSO, or different concentrations of the RBBP9 SH inhibitor ML114 (Key Organics, Cornwall, UK) in 0.25% DMSO. Unless otherwise stated, treatments were added 72 h after cell seeding to ensure maximal cell yield of treated hPSCs for downstream assays. In all cases, the final DMSO concentration was 0.25%. To assess recovery of hPSCs after ML114 treatment, hPSCs were harvested on Day 7 of treatment and re-seeded into optimal hPSC maintenance conditions and maintained via standard passaging for up to 12 weeks. Cell counts were recorded at each passage prior to hPSC re-seeding.

### 4.2. Colony-Forming Cell Assay

The pluripotent CFC assay was performed as previously described [29]. Briefly, single cell suspensions of hPSCs were plated in mTeSR1 and treated with 0, 5, 50 and 100 μM ML114 for 7 days. The culture dishes were processed at 1, 2, and 5 days after treatment, using the Alkaline Phosphatase Leukocyte Kit (Sigma Aldrich) as per the manufacturer’s instructions. Colony numbers were counted manually using an Olympus CKX41 inverted microscope, with images captured using a digital camera and Q Capture Pro v6 software. The cell number per colony was determined by approximating the plate area occupied by each colony, based on the estimated average colony diameters. The Student’s *t*-test was used to determine statistical significance.

### 4.3. Dead Cell Counting

Cell culture media was collected separately for each treatment. The treated cells were then washed with PBS and the PBS was combined with the appropriate media sample. These media samples were then centrifuged (300× *g*, 5 min) before the number of dead cells present in each sample was manually counted using a hemocytometer.

### 4.4. Flow Cytometry Analysis of Pluripotency Markers

hPSCs were harvested as single cell suspensions, fixed with 2% para-formaldehyde, then stored in 10% FBS in PBS. For detection of extracellular antigens, hPSCs were kept in 10% FBS in PBS. For detection of intracellular antigens, hPSCs were resuspended in 0.1% saponin permeabilization buffer in 10% FBS in PBS for 15 min [15,51]. hPSCs were incubated for 20 min with the following primary antibodies: 2 μg/μL anti-TRA-1-60 and 2 μg/μL anti-TRA-1-81 (Abcam, Cambridge, UK); or 0.25 μg/μL anti-OCT4 antibody (BD Biosciences). The anti-TRA antibodies were detected using Alexa Fluor-488 anti-mouse IgM secondary antibody (Thermo Fisher Scientific, Sydney, Australia), and the anti-OCT4 antibody using Alexa Fluor-488 anti-mouse IgG antibody (Thermo Fisher Scientific). Samples were analyzed using a MACSQuant^®^ Analyser Flow Cytometer (Miltenyi Biotec, North Ryde, Australia). Biological replicates for each sample were averaged, and comparisons were made using the Student’s *t*-test.

### 4.5. Cell Proliferation Assay

Prior to confluence, ML114-treated hPSCs were incubated for 2 h or 6 h with 10 μM EdU label (Thermo Fisher Scientific). The cells were then harvested as a single cell suspension and fixed with 4% para–formaldehyde in PBS for 15 min. Samples were then washed with 1% BSA in PBS, centrifuged (300× *g*, 1 min), and the cell pellet processed using the Click-iT EdU detection kit (Thermo Fisher Scientific) as per the manufacturer’s instructions. The EdU labelled samples were then analyzed via flow cytometry, and comparisons were made using the Student’s *t*-test.

### 4.6. Teratoma and Karyotyping Analyses

hPSCs were treated with 100 μM ML114 in 0.25% DMSO for 7 days. The cells were then harvested and re-seeded as single cell suspensions in mTeSR1™ and passaged for a further 11 weeks. Assessment of teratoma formation was performed externally by the StemCore Facility, University of Queensland, with histological analysis of haematoxylin and eosin-stained sections performed by an expert pathologist. Karyotyping was also performed by the StemCore Facility.

### 4.7. Affymetrix, Gene Ontology/Promoter Analyses, and Real-Time PCR

Affymetrix profiling was performed on 6 hPSC samples (3 × DMSO-treated and 3 × 100 μM ML114-treated). Each sample was harvested after 7 days of treatment for RNA, and 500 ng of RNA was analyzed using HuGene-1.0-st-v1 arrays (Affymetrix, Santa Clara, CA, USA) at the Ramaciotti Centre for Gene Function Analysis (University of New South Wales, Sydney, Australia). Analysis of Affymetrix profiling data was performed using the GenePattern software suite [52] to determine transcripts as present (P) or absent (A) across each treatment. Comparison between control- and ML114-treated samples identified genes with statistically-significant changes in expression (*p* < 0.05; false discovery rate <0.05). Genes commonly affected by loss of RBBP9 activities were identified by comparing genes significantly affected by ML114- or siRNA treatment [12]. Gene ontology analyses were performed using the DAVID Functional Analysis Tool [53,54,55]. Predictions of regulating transcription factors were performed with the PASTAA online suite [56], using both proximal promoter analyses (±400 base pairs on either side of the transcription start sites) and distal analyses (10,000 kilo bases upstream from the transcription start sites). Candidate regulatory transcription factors were identified with *p* value < 0.05. For real-time PCR, RNA was purified using the Bioline Isolate II RNA purification kit (Bioline, Eveleigh, Australia). cDNA was synthesized using: 500 ng of purified RNA; random hexamer primers; dNTPs; RNase inhibitor; buffer; Bioscript reverse transcriptase (all from Bioline); and a Mastercycler (42 °C for 60 min; then 70 °C for 10 min). Real-time PCR was performed using an Mx3005P qPCR system (Aligent technologies, Sydney, Australia) with MxPro software, and consisted of 40 cycles of: denaturation (95 °C, 5.5 min); annealing (55–60 °C, 20 s); and extension (72 °C, 2.5 min). Forward and reverse NFYA primers were: GCACGGAGTGTACCTCACAG/TGCTGTATACTGCTCCATGGTC. Forward and reverse GAPDH primers were: ATTGCCCTCAACGACCACT/ATGAGGTCCACCACCCTGT. Triplicate cycle threshold values were analyzed using the Pfaffl method [57] and the Student’s *t*-test performed to assess differences in gene expression.

### 4.8. SDS-PAGE and Western Blotting

Treated hPSCs were harvested as a single cell suspension using TryPLE (Gibco, Sydney, Australia) and protein extracted in protein lysis buffer (25 mM Tris, 150 mM NaCl, 1 mM EDTA, 1 mM EGTA, 1% Triton-X 100, 1 mM Na Vanadate, 1 mM PMSF, 5 μg Aprotinin, protease inhibitors, pH 7.4). Samples underwent a 4M urea exchange purification and concentration step using a 3.000 Da molecular weight cut-off filter (Merck Millipore, Bayswater, Australia). Samples were quantified using an EZQ Protein Quantification assay. For electrophoresis, samples were heated in SDS loading/sample buffer for 5 min prior to loading into one-dimensional SDS-PAGE gels. The electrophoresis was performed using 25 μg of protein per sample, using 12% Mini Protean^®^ TGX pre-cast gels (Bio-Rad Laboratories, Gladesville, Australia). Gels were stained for total protein with Neuhoff Coomassie stain (0.1% CBB-G250, 2% phosphoric acid, 10% ammonium sulfate, and 20% MeOH) followed by 5 × 5 min washes in 0.5 M NaCl [58]. Gels were imaged using a FLA-9000 imager (Fuji Film) using 685 nm laser excitation and ≥750 nm emission with scanning pixel size of 100 μm [59]. For Western blotting, proteins were transferred onto 0.2 μM PVDF membranes (Merck Millipore) using 120 V, 4 °C for 1 h. Membranes were blocked and then probed overnight at 4 °C with 1/500 of primary antibody to detect DEAF-1 (Sapphire Bioscience, Redfern, Australia); NFYA (Sapphire Bioscience, Sydney, Australia); and GAPDH (Sapphire Bioscience). Membranes were then washed, probed with secondary antibodies at room temperature for 1–2 h, washed again. Blots were imaged using Luminata Cresendo Western HRP Substrate (Merck Millipore) using a LAS-4000 with Multi-Gauge v3.0 software (Fuji Film). Entire Western blot images were analyzed using ImageJ software.

### 4.9. Statistical Analyses

For comparison of two conditions, the Students *t*-test was used. Where three or more treatments were compared, analysis of variance (ANOVA) was used. Where ANOVA analysis determined a significant difference (*p* > 0.5), individual post hoc *t*-tests were performed to identify specific treatments that differed from the control treatment.

## Figures and Tables

**Table 1:** Proximal analysis (± 400 bp) of genes affected by ML114 in hPSCs.

*Matrix*	*Transcription Factor*	*Association Score*	*p-Value*
ARNT_02	Arnt	10.876	0.00 × 10
STRA13_01	Stra13	9.882	0.00 × 10
**NFY_01**	**N/A**	**7.035**	**5.00 × 10^−6^**
TFIII_Q6	Tfii-i	6.654	1.70 × 10^−5^
MAZR_01	Mazr	6.294	4.20 × 10^−5^
NFKAPPAB65_01	Rela	6.156	5.40 × 10^−5^
USF_Q6	Usf1, Usf2a	6.139	5.40 × 10^−5^
MZF1_01	Mzf-1	6.09	6.70 × 10^−5^
**DEAF1_01**	**Deaf-1**	**5.999**	**8.00 × 10^−5^**
USF_Q6_01	Usf-1, Usf1	5.802	1.27 × 10^−4^
ATF1_Q6	Atf-1	5.652	1.63 × 10^−4^
NERF_Q2	Nerf-1a	5.422	2.79 × 10^−4^
**DEAF1_02**	**Deaf-1**	**5.354**	**3.16 × 10^−4^**
MTF1_Q4	Mtf-1	5.266	3.98 × 10^−4^
MOVOB_01	Movo-b	5.161	4.91 × 10^−4^
EGR2_01	Egr-2	5.155	4.94 × 10^-4^
CETS1P54_03	C-ets-1	4.928	8.23 × 10^−4^
NRF2_01	N/A	4.768	1.17 × 10^−3^
ARNT_01	Arnt	4.584	1.72 × 10^−3^
EGR3_01	Egr-3	4.565	1.80 × 10^−3^
YY1_Q6_02	Yy1	4.563	1.80 × 10^−3^

**Table 2:** Distal analysis (± −10 kb) of genes affected by ML114 in hPSCs.

*Matrix*	*Transcription Factor*	*Association Score*	*p-Value*
**NFY_01**	**N/A**	**4.681**	**1.37 × 10^−3^**
**ETS_Q4**	**Erf, Elf-1**	**4.194**	**3.76 × 10^−3^**
NFKAPPAB50_01	N/A	4.147	4.05 × 10^−3^
PR_02	N/A	4.059	4.91 × 10^−3^
**NFY_Q6**	**Cbf-a, Cbf-b**	**3.616**	**1.21 × 10^−2^**
SZF11_01	N/A	3.597	1.26 × 10^−2^
**NFY_Q6_01**	**Cbf-a, Cbf-b**	**3.515**	**1.47 × 10^−2^**
**DEAF1_02**	**Deaf-1**	**3.393**	**1.88 × 10^−2^**
**NFY_C**	**Cbf-a, Cbf-b**	**3.314**	**2.15 × 10^−2^**
CRX_Q4	Crx, Rx	3.311	2.17 × 10^−2^
ETS2_B	C-ets-1, C-ets-2	3.293	2.27 × 10^−2^
MAZR_01	Mazr	3.288	2.34 × 10^−2^
OCT1_01	Pou2f1, Pou2f1a	3.19	2.82 × 10^−2^
E2F_Q4_01	Dp-1, E2f-1	3.149	3.07 × 10^−2^
CDP_02	Cutl1	3.037	3.71 × 10^−2^
CLOX_01	Cutl	3.037	3.71 × 10^−2^
YY1_02	Yy1	3.014	3.98 × 10^−2^
HNF4_Q6_02	Hnf-4, Hnf-4alpha	2.98	4.20 × 10^−2^
HSF1_Q6	Hsf1, Hsf1long	2.898	4.81 × 10^−2^

**Table 3:** Proximal analysis (± 400 bp) of genes commonly affected by siRNA SH inhibition and ML114 treatment.

*Matrix*	*Transcription Factor*	*Association Score*	*p-Value*
LBP1_Q6	N/A	5.55	1.12 × 10^−4^
PAX4_03	Pax-4a	4.392	1.32 × 10^−3^
MAZR_01	Mazr	4.348	1.43 × 10^−3^
HEN1_01	N/A	4.133	2.10 × 10^−3^
PAX9_B	Pax-9a	4.133	2.10 × 10^−3^
ETF_Q6	N/A	4.132	2.10 × 10^−3^
HEB_Q6	Heb	3.898	3.45 × 10^−3^
E2_Q6	N/A	3.806	4.25 × 10^−3^
AP4_Q6_01	Ap-4	3.804	4.25 × 10^−3^
GC_01	N/A	3.556	7.05 × 10^−3^
**DEAF1_01**	**Deaf-1**	**3.535**	**7.34 × 10^−3^**
**SP1_Q4_01**	**Sp1, Sp2**	**3.468**	**8.12 × 10^−3^**
**SP1_Q6**	**Sp1**	**3.468**	**8.12 × 10^−3^**
**SP1_Q6_01**	**Sp1, Sp3**	**3.468**	**8.12 × 10^−3^**
PAX5_01	Pax-5	3.425	9.26 × 10^−3^
**SP1_01**	**Sp1**	**3.357**	**1.03 × 10^−2^**
MYB_Q6	C-myb	3.352	1.03 × 10^−2^
GATA1_01	Gata-1	3.349	1.03 × 10^−2^
E2_01	N/A	3.259	1.25 × 10^−2^
CHCH_01	Chch	3.126	1.62 × 10^−2^

**Table 4:** Distal analysis (± −10 kb) of genes commonly affected by siRNA SH inhibition and ML114 treatment.

*Matrix*	*Transcription Factor*	*Association Score*	*p-Value*
POU1F1_Q6	Pou1f1, Pou1f1a	4.809	5.40 × 10^−4^
YY1_02	Yy1	3.697	5.30 × 10^−4^
AHR_01	Ahr	3.413	9.58 × 10^−3^
PU_Q6	Pu.1	3.365	1.01 × 10^−2^
LXR_DR4_Q3	N/A	3.153	1.55 × 10^−2^
CDP_02	Cut1	3.061	1.83 × 10^−2^
GATA3_03	Gata-3	3.043	1.92 × 10^−2^
NFKAPPAB50_01	N/A	3.017	2.07 × 10^−2^
STAT3_02	Stat3	2.990	2.20 × 10^−2^
ROAZ_01	Roaz	2.969	2.27 × 10^−2^
COUP_DR1_Q6	Coup-tf1, Coup-tf2	2.931	2.41 × 10^−2^
GATA1_02	Gata-1	2.893	2.59 × 10^−2^
NFE2_01	Nf-e2	2.799	3.09 × 10^−2^
OLFA_01	Olf-1	2.772	2.23 × 10^−2^
HFH4_01	Foxf1, Foxj1	2.750	3.34 × 10^−2^
LYF1_01	N/A	2.748	3.35 × 10^−2^
E2_Q6	N/A	2.720	3.67 × 10^−2^
NFMUE1_Q6	N/A	2.699	3.82 × 10^−2^
PBX1_02	Pbx1a	2.646	4.14 × 10^−2^
**CBF_01**	**N/A**	**2.645**	**4.14 × 10^−2^**

## Supplementary Materials

Supplementary Materials can be found at https://www.mdpi.com/1422-0067/21/23/8983/s1.

## Abbreviations

hPSC	Human pluripotent stem cell
RBBP9	Retinoblastoma binding protein 9
SH	Serine hydrolase
siRNA	Small interfering RNA
CFC	Colony-forming cell
