# Supplementary material for: Application of the RBBP9 Serine Hydrolase Inhibitor, ML114, Decouples Human Pluripotent Stem Cell Proliferation and Differentiation
Source: Int J Mol Sci. 2020 Nov 26;21(23):8983. doi: 10.3390/ijms21238983 (PMC7730578; doi:10.3390/ijms21238983)

Supplementary Material

**
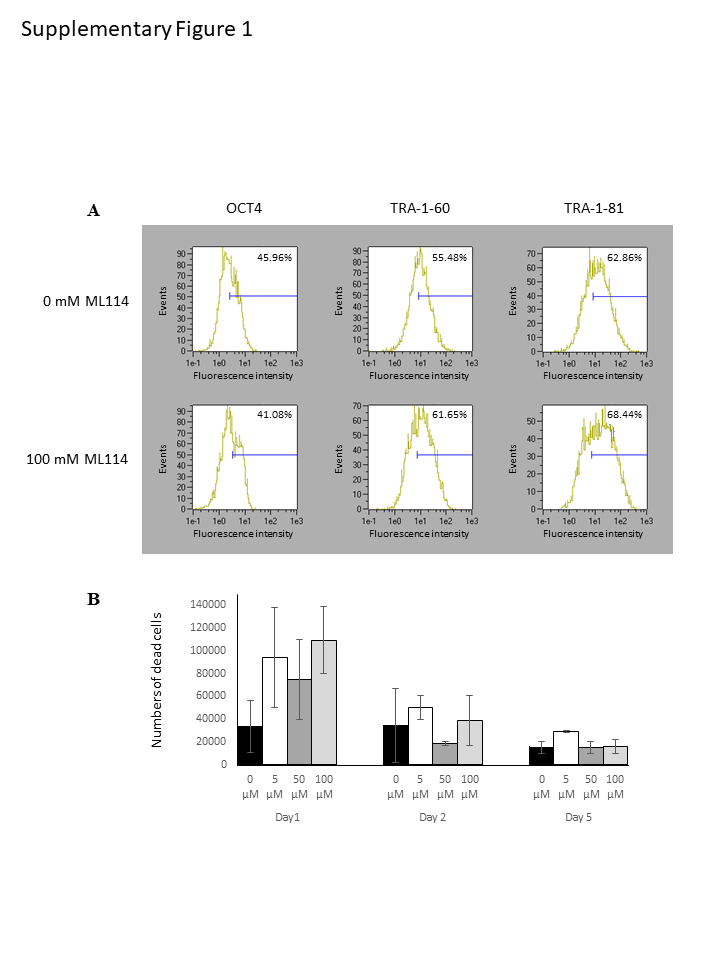
Supplementary Figure 1.** Counts of dead cell numbers showed no significant difference between the treatments after 24 hours (Day 1; p = 0.1), 48 hours (Day 2; p = 0.75) or 120 hours (Day 5; p = 0.24).

**
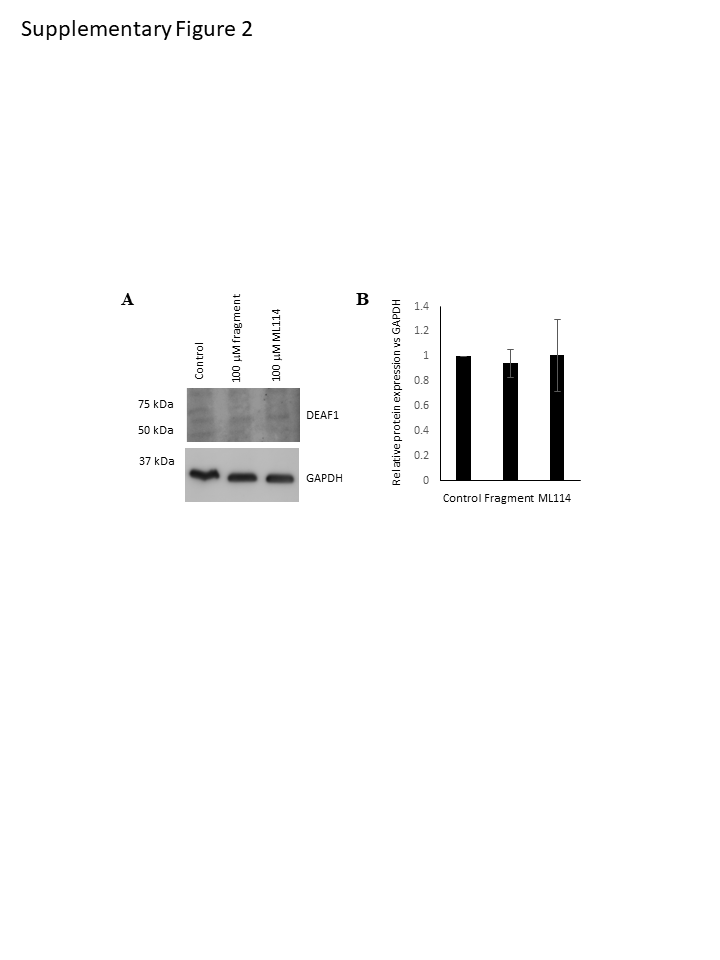
Supplementary Figure 2. DEAF1 protein expression is not significantly increased by ML114.** **A, B)** Western blot (A) and associated densitometry quantification (B) showing no difference in DEAF1 protein levels between hPSCs treated with Control (DMSO), ML114 fragment and ML114 (n = 3 for all treatments).


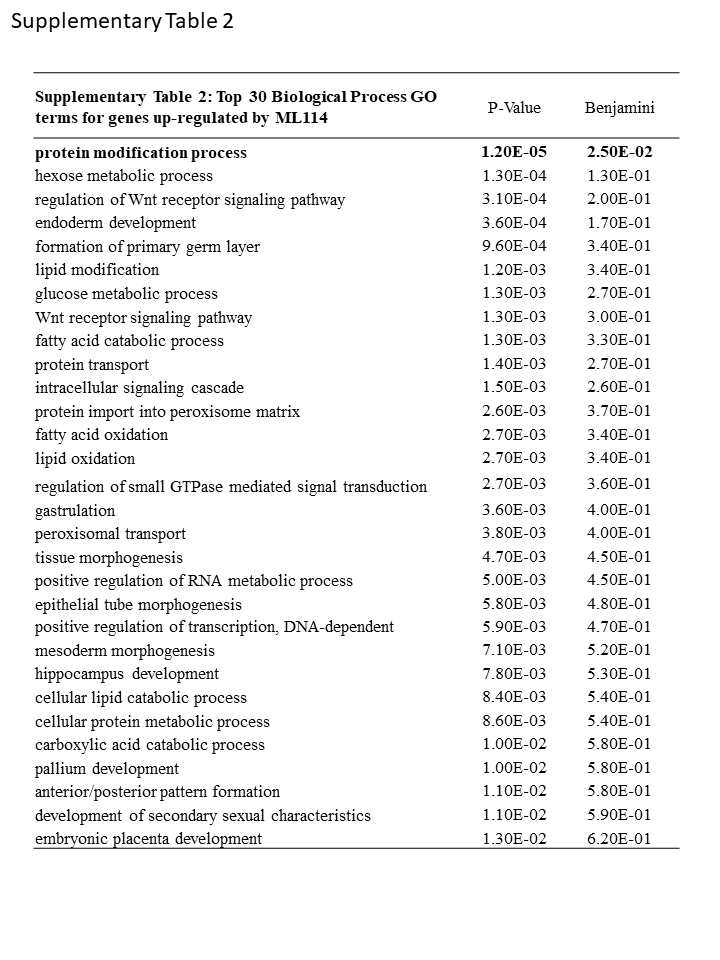

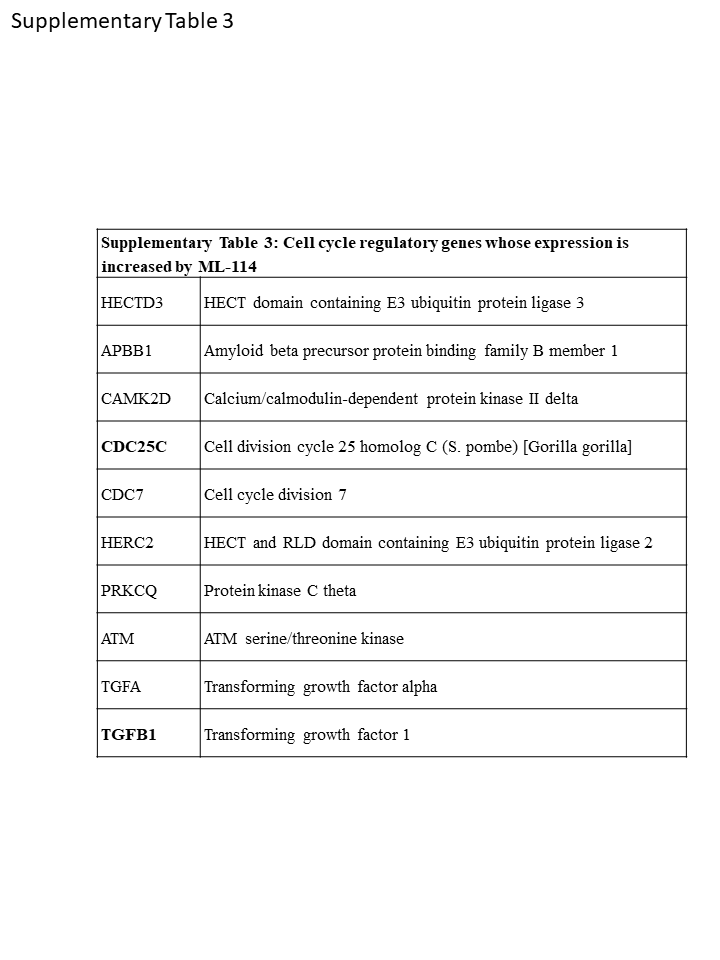


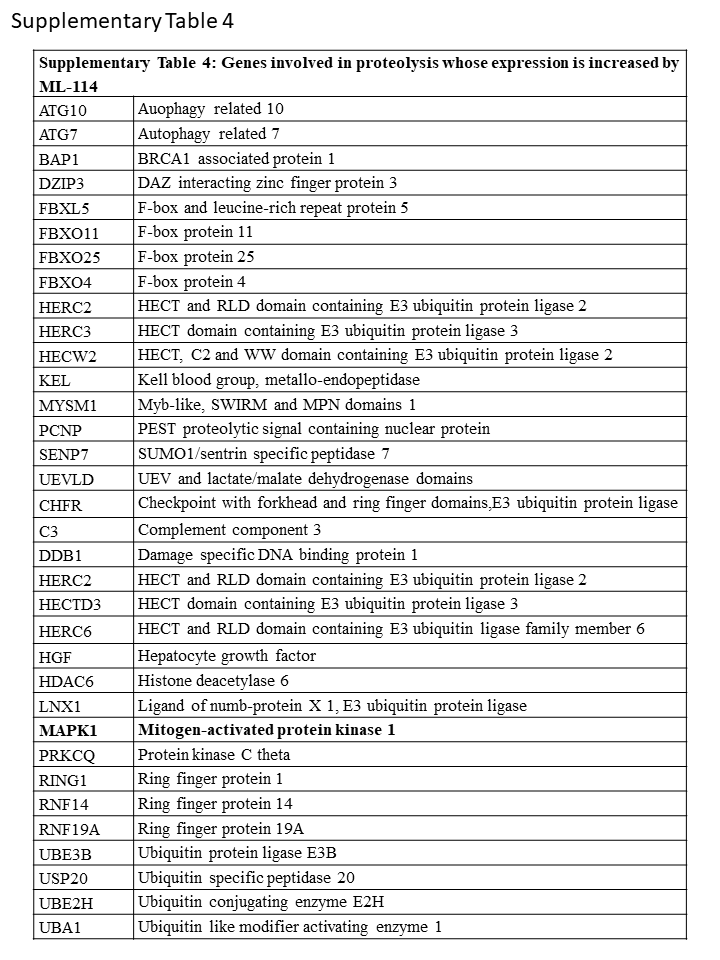


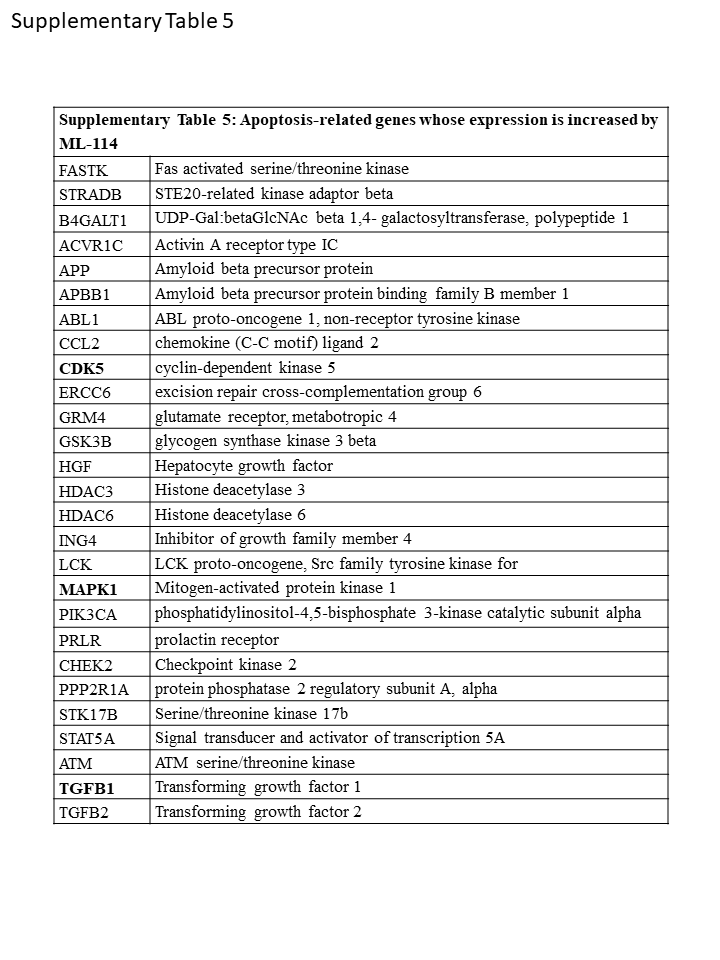

Supplement: Supplementary file 1 [file ijms-21-08983-s001.zip › Supplementary material R2.docx]
