# Supplementary material for: Application of the RBBP9 Serine Hydrolase Inhibitor, ML114, Decouples Human Pluripotent Stem Cell Proliferation and Differentiation
Source: Int J Mol Sci. 2020 Nov 26;21(23):8983. doi: 10.3390/ijms21238983 (PMC7730578; doi:10.3390/ijms21238983)
Supplement: Supplementary file 1 [file ijms-21-08983-s001.zip › Supplementary material R2.pdf]

Supplementary Material

Supplementary Figure 1

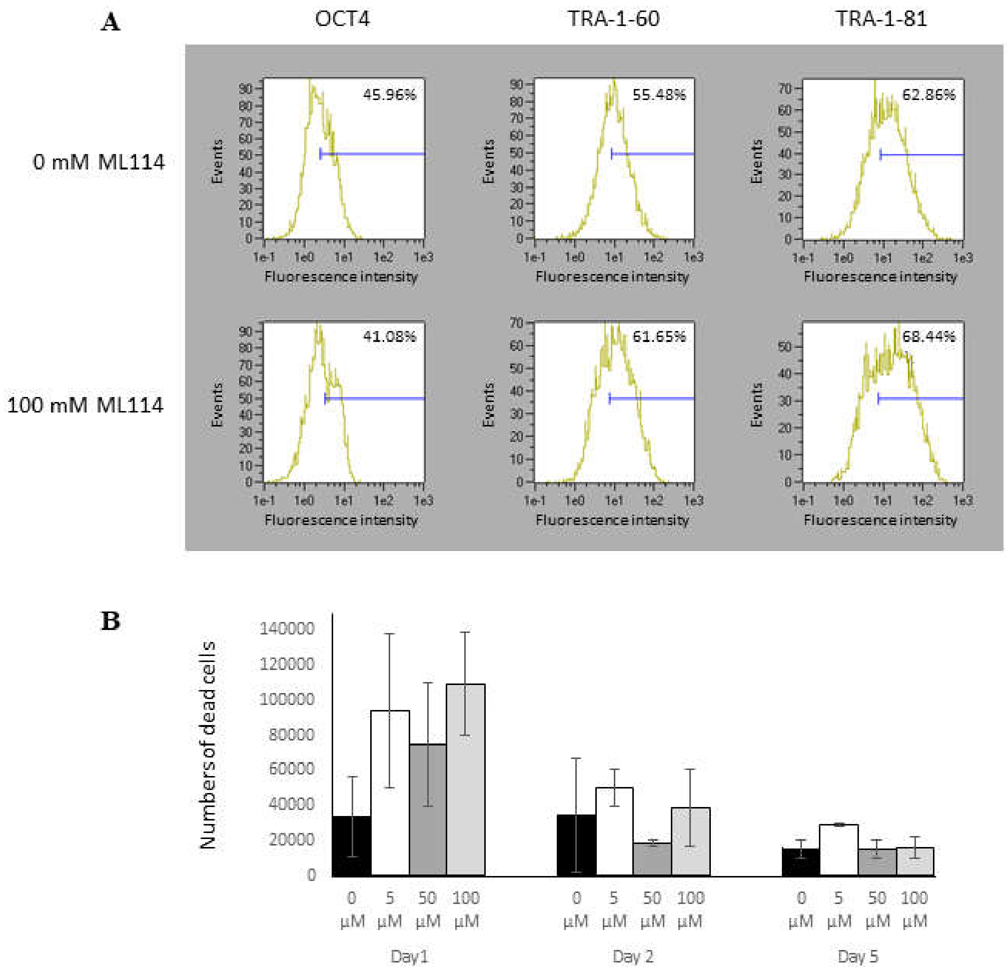

Supplementary Figure 1. Counts of dead cell numbers showed no significant difference between the treatments after 24 hours (Day 1; p = 0.1), 48 hours (Day 2; p = 0.75) or 120 hours (Day 5; p = 0.24).

Supplementary Figure 2

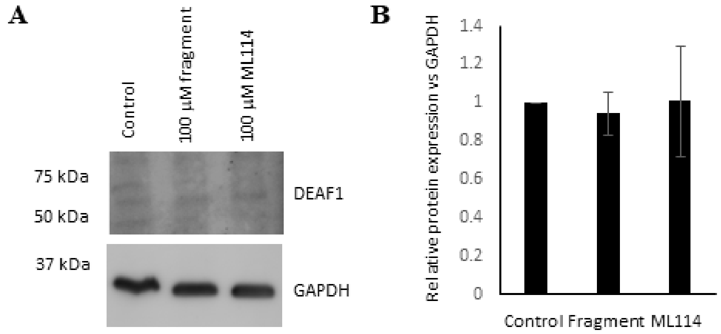

Supplementary Figure 2. DEAF1 protein expression is not significantly increased by ML114. A, B) Western blot (A) and associated densitometry quantification (B) showing no difference in DEAF1 protein levels between hPSCs treated with Control (DMSO), ML114 fragment and ML114 (n = 3 for all treatments).

Supplementary Table 2

**Supplementary Table 2: Top 30 Biological Process GO terms for genes up-regulated by ML114**	P-Value	Benjamini
**protein modification process**	**1.20E-05**	**2.50E-02**
hexose metabolic process	1.30E-04	1.30E-01
regulation of Wnt receptor signaling pathway	3.10E-04	2.00E-01
endoderm development	3.60E-04	1.70E-01
formation of primary germ layer	9.60E-04	3.40E-01
lipid modification	1.20E-03	3.40E-01
glucose metabolic process	1.30E-03	2.70E-01
Wnt receptor signaling pathway	1.30E-03	3.00E-01
fatty acid catabolic process	1.30E-03	3.30E-01
protein transport	1.40E-03	2.70E-01
intracellular signaling cascade	1.50E-03	2.60E-01
protein import into peroxisome matrix	2.60E-03	3.70E-01
fatty acid oxidation	2.70E-03	3.40E-01
lipid oxidation	2.70E-03	3.40E-01
regulation of small GTPase mediated signal transduction	2.70E-03	3.60E-01
gastrulation	3.60E-03	4.00E-01
peroxisomal transport	3.80E-03	4.00E-01
tissue morphogenesis	4.70E-03	4.50E-01
positive regulation of RNA metabolic process	5.00E-03	4.50E-01
epithelial tube morphogenesis	5.80E-03	4.80E-01
positive regulation of transcription, DNA-dependent	5.90E-03	4.70E-01
mesoderm morphogenesis	7.10E-03	5.20E-01
hippocampus development	7.80E-03	5.30E-01
cellular lipid catabolic process	8.40E-03	5.40E-01
cellular protein metabolic process	8.60E-03	5.40E-01
carboxylic acid catabolic process	1.00E-02	5.80E-01
pallium development	1.00E-02	5.80E-01
anterior/posterior pattern formation	1.10E-02	5.80E-01
development of secondary sexual characteristics	1.10E-02	5.90E-01
embryonic placenta development	1.30E-02	6.20E-01

Supplementary Table 3

**Supplementary Table 3: Cell cycle regulatory genes whose expression is increased by ML-114**	
HECTD3	HECT domain containing E3 ubiquitin protein ligase 3
APBB1	Amyloid beta precursor protein binding family B member 1
CAMK2D	Calcium/calmodulin-dependent protein kinase II delta
**CDC25C**	Cell division cycle 25 homolog C (S. pombe) [Gorilla gorilla]
CDC7	Cell cycle division 7
HERC2	HECT and RLD domain containing E3 ubiquitin protein ligase 2
PRKCQ	Protein kinase C theta
ATM	ATM serine/threonine kinase
TGFA	Transforming growth factor alpha
**TGFB1**	Transforming growth factor 1

Supplementary Table 4

**Supplementary Table 4: Genes involved in proteolysis whose expression is increased by ML-114**	
ATG10	Auophagy related 10
ATG7	Autophagy related 7
BAP1	BRCA1 associated protein 1
DZIP3	DAZ interacting zinc finger protein 3
FBXL5	F-box and leucine-rich repeat protein 5
FBXO11	F-box protein 11
FBXO25	F-box protein 25
FBXO4	F-box protein 4
HERC2	HECT and RLD domain containing E3 ubiquitin protein ligase 2
HERC3	HECT domain containing E3 ubiquitin protein ligase 3
HECW2	HECT, C2 and WW domain containing E3 ubiquitin protein ligase 2
KEL	Kell blood group, metallo-endopeptidase
MYSM1	Myb-like, SWIRM and MPN domains 1
PCNP	PEST proteolytic signal containing nuclear protein
SENP7	SUMO1/sentrin specific peptidase 7
UEVLD	UEV and lactate/malate dehydrogenase domains
CHFR	Checkpoint with forkhead and ring finger domains,E3 ubiquitin protein ligase
C3	Complement component 3
DDB1	Damage specific DNA binding protein 1
HERC2	HECT and RLD domain containing E3 ubiquitin protein ligase 2
HECTD3	HECT domain containing E3 ubiquitin protein ligase 3
HERC6	HECT and RLD domain containing E3 ubiquitin ligase family member 6
HGF	Hepatocyte growth factor
HDAC6	Histone deacetylase 6
LNX1	Ligand of numb-protein X 1, E3 ubiquitin protein ligase
**MAPK1**	**Mitogen-activated protein kinase 1**
PRKCQ	Protein kinase C theta
RING1	Ring finger protein 1
RNF14	Ring finger protein 14
RNF19A	Ring finger protein 19A
UBE3B	Ubiquitin protein ligase E3B
USP20	Ubiquitin specific peptidase 20
UBE2H	Ubiquitin conjugating enzyme E2H
UBA1	Ubiquitin like modifier activating enzyme 1

Supplementary Table 5

**Supplementary Table 5: Apoptosis-related genes whose expression is increased by ML-114**	
FASTK	Fas activated serine/threonine kinase
STRADB	STE20-related kinase adaptor beta
B4GALT1	UDP-Gal:betaGlcNAc beta 1,4- galactosyltransferase, polypeptide 1
ACVR1C	Activin A receptor type IC
APP	Amyloid beta precursor protein
APBB1	Amyloid beta precursor protein binding family B member 1
ABL1	ABL proto-oncogene 1, non-receptor tyrosine kinase
CCL2	chemokine (C-C motif) ligand 2
**CDK5**	cyclin-dependent kinase 5
ERCC6	excision repair cross-complementation group 6
GRM4	glutamate receptor, metabotropic 4
GSK3B	glycogen synthase kinase 3 beta
HGF	Hepatocyte growth factor
HDAC3	Histone deacetylase 3
HDAC6	Histone deacetylase 6
ING4	Inhibitor of growth family member 4
LCK	LCK proto-oncogene, Src family tyrosine kinase for
**MAPK1**	Mitogen-activated protein kinase 1
PIK3CA	phosphatidylinositol-4,5-bisphosphate 3-kinase catalytic subunit alpha
PRLR	prolactin receptor
CHEK2	Checkpoint kinase 2
PPP2R1A	protein phosphatase 2 regulatory subunit A, alpha
STK17B	Serine/threonine kinase 17b
STAT5A	Signal transducer and activator of transcription 5A
ATM	ATM serine/threonine kinase
**TGFB1**	Transforming growth factor 1
TGFB2	Transforming growth factor 2
